# Turbulent kinetic energy in the ascending aorta is greater in bicuspid than tricuspid aortic valve stenosis

**DOI:** 10.1186/1532-429X-17-S1-O88

**Published:** 2015-02-03

**Authors:** Margaret Loudon, Malenka M Bissell, Petter Dyverfeldt, Carl Johan Carlhall, Tino Ebbers, Aaron T Hess, Bernard D Prendergast, Stefan Neubauer, Saul G Myerson

**Affiliations:** 1 University of Oxford Centre for Clinical Magnetic Resonance Research (OCMR), Cardiovascular Medicine, University of Oxford, Oxford, UK; 2 Center for Medical Image Science and Visualization (CMIV), Linköping University, Linköping University, Sweden; 3 Department of Cardiology, Oxford University NHS Trust, Oxford, UK

## Background

The main determinant of the haemodynamic significance of aortic stenosis (AS) is the irreversible pressure loss that is created by the stenosis. The majority of the pressure loss is caused by conversion/dissipation of turbulent kinetic energy (TKE) to heat. Recent developments in cardiac magnetic resonance 4D flow imaging have allowed the non-invasive assessment of TKE. Bicuspid aortic valve disease is known to be associated with a larger ascending aorta and disordered flow patterns, and we hypothesised that peak TKE would be higher in bicuspid AS than tricuspid AS.

## Methods

15 patients with bicuspid AS (mean age 63.6 years; mean aortic valve area 1.4cm^2^; mean dimension of the ascending aorta at the level of the pulmonary artery 3.9cm) and no more than mild other valve disease were compared with 22 patients with tricuspid AS (mean age 72.9 years; mean aortic valve area 1.2cm^2^; mean dimension of the ascending aorta at the level of the pulmonary artery 3.2cm) and no more than mild other valve disease.

All subjects underwent time resolved, three dimensional cine magnetic resonance flow imaging at 3 Tesla, for the assessment of peak TKE. The peak TKE was obtained by integrating the TKE per voxel across the ascending aorta at each time frame of the cardiac cycle.

## Results

**Figure 1:**
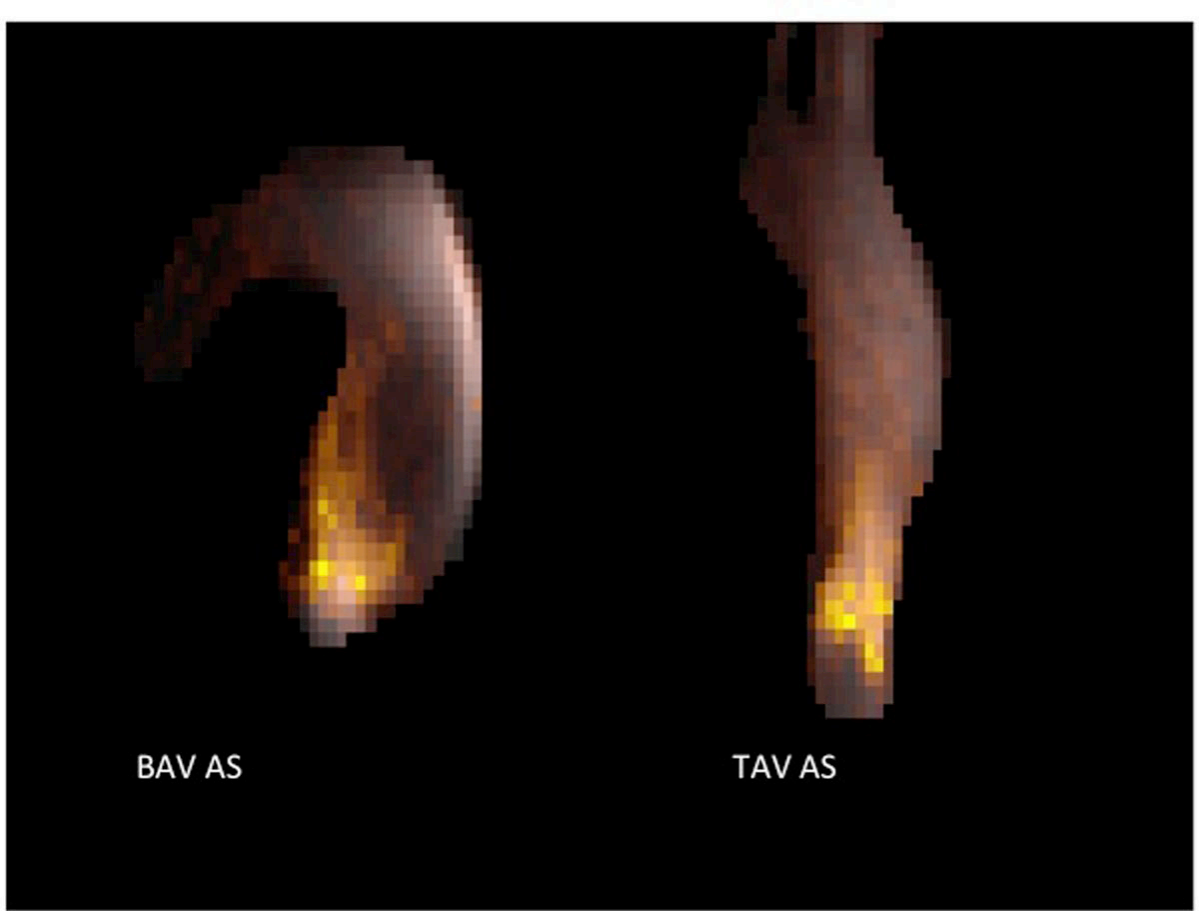


On visual inspection, the highest TKE was seen at valvular level and dispersed centrally in tricuspid AS. In bicuspid AS the highest TKE is seen at valvular level, then as a jet hitting the wall of the proximal aorta, before dispersing (see Figure 1).

Peak TKE was significantly higher in the bicuspid AS patients compared to tricuspid AS patients (see table).

**Table 1:** Peak TKE

	Bicuspid AS	Tricuspid AS	p-value (unpaired t-test)
Peak TKE- valve and ascending aorta	28.5mJ (±12.6)	19.6mJ (±8.0)	0.03

Peak TKE- Ascending aorta alone (sinuses of Valsalva to the arch)	18.9mJ (±8.4)	11.0mJ (±4.1)	0.006

Values are mean peak TKE in mJ (±standard deviation)

## Conclusions

Bicuspid AS is associated with significantly higher peak TKE compared with tricuspid AS of comparable severity. This applies both when including the valve, and when measured in the ascending aorta alone, and may result from the larger aorta and disordered flow patterns typically seen in bicuspid AS. This implies that bicuspid AS may be associated with a greater haemodynamic burden for similar degrees of stenosis.

## Funding

The research was supported by the National Institute for Health Research (NIHR) Oxford Biomedical Research Centre based at The Oxford University Hospitals Trust at the University of Oxford and the Britsh Heart Foundation, the Swedish Research Council, the Swedish Heart-Lung Foundation.

